# Temporal induction of the homeodomain transcription factor Nkx2-1 is sufficient to respecify foregut and hindgut endoderm to a pulmonary fate in *Xenopus laevis*

**DOI:** 10.17912/micropub.biology.001610

**Published:** 2025-05-07

**Authors:** Brian A Hyatt, Erin Lundberg, Rachael Eye, Scott A Rankin, Aaron M Zorn

**Affiliations:** 1 Biological Sciences, Bethel University, Saint Paul, Minnesota, United States; 2 Center for Stem Cell and Organoid Medicine (CuSTOM), Division of Developmental Biology, Perinatal Institute, Cincinnati Children's Hospital Medical Center, Cincinnati, Ohio, United States; 3 College of Medicine, Department of Pediatrics, University of Cincinnati

## Abstract

The ability of transcription factors (TFs) to regulate cell fate decisions is paramount in developmental, homeostatic, and pathogenic contexts. The homeodomain TF NKX2-1 is an essential and evolutionarily conserved master regulator of pulmonary fate in vertebrates. In this study, we tested the spatial-temporal ability of Xenopus and Human NKX2-1 to respecify foregut and hindgut endoderm in developing *Xenopus laevis* embryos into a pulmonary fate, as indicated by expression of pulmonary surfactant genes *sftpc* and *sftpb* . Interestingly, we find that both Human and Xenopus NKX2-1 can induce the ectopic expression of pulmonary surfactant genes in foregut and hindgut endoderm over a wide range of developmental times, as well as suppress the expression of midgut and hindgut specific genes. These results suggest a single pulmonary TF can reprogram developing endoderm and specify pulmonary fate. In addition, our work provides a comparative platform for future studies investigating how mutations in Human *NKX2-1* may affect its transcriptional activity.

**Figure 1. Spatial-temporal overexpression of Xenopus and Human Nkx2-1 results in ectopic pulmonary fate and suppressed midgut and hindgut fates:**
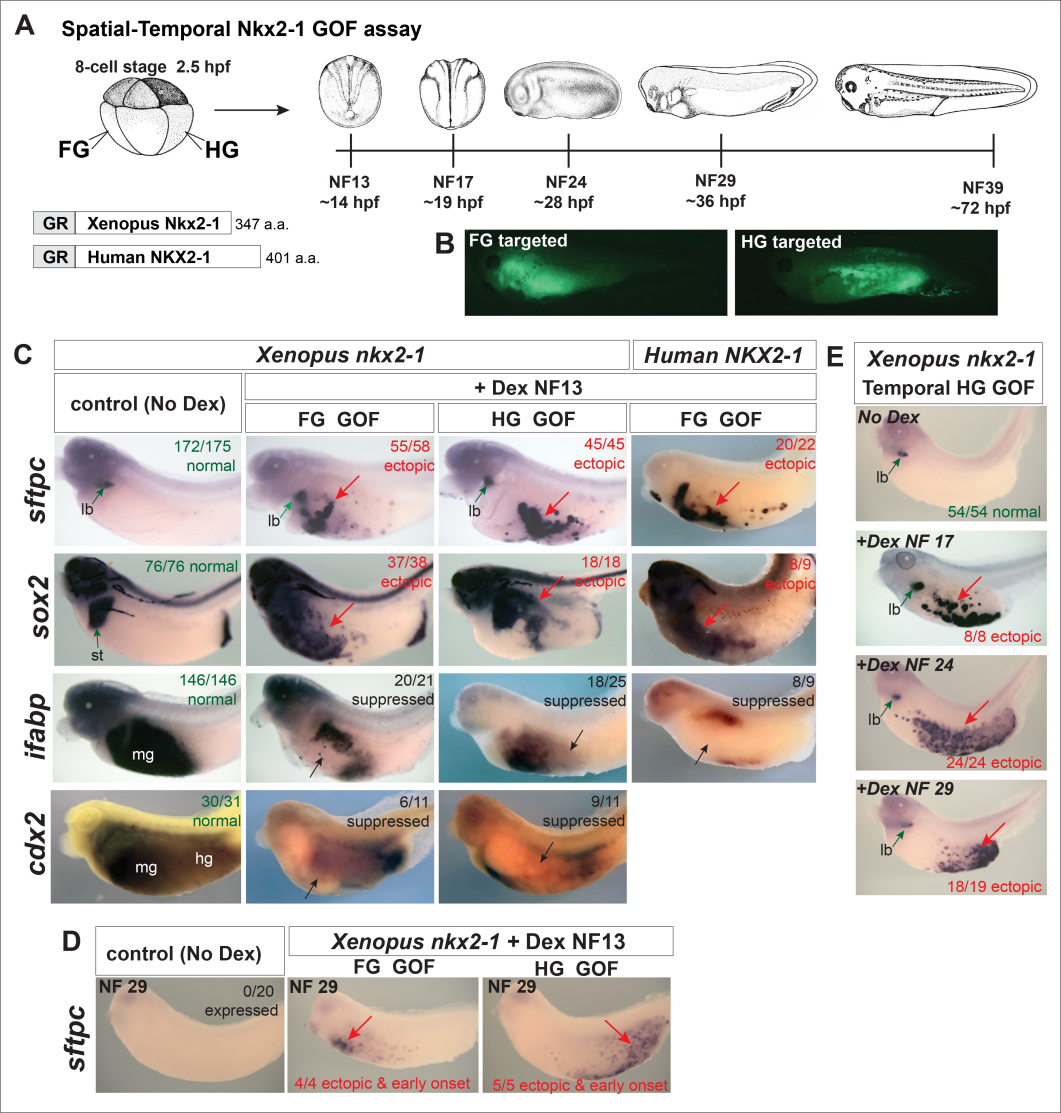
**(A)** Experimental schematic of Nkx2-1 Gain of function (GOF) assay in Xenopus embryos. 400pg of mRNA encoding GR-fusions of Xenopus Nkx2-1 and Human NKX2-1 was co-injected with 50pg GFP mRNA into dorsal-vegetal (targeting FG) or ventral vegetal blastomeres (targeting HG) blastomeres at the 8-cell stage, and embryos were transferred into culture buffer containing Dexamethasone (Dex) at the indicated stages to induce nuclear Nkx2-1 activity. ( **B)** GFP fluorescence images showing representative FG and HG targeting. ( **C-E** ) *In situ* hybridization analysis of experimental embryos. Representative staining patterns are shown for each condition with the numbers of embryos displaying that pattern listed; numbers are the sum of 4 combined injection experiments. Green arrows indicate normal endogenous expression of the gene assayed, red arrows indicate ectopic expression domains, and black arrows indicate reduced expression. ( **C** ) Analysis at NF39 for the indicated genes after Dex induction at NF13: pulmonary specific marker *sftpc* , *sox2* marks foregut/esophogeal and stomach (st) endoderm, *ifabp* marks midgut endoderm, and *cdx2* marks mid/hindgut endoderm *.* **(D)** Analysis of *sftpc* expression at NF29, before the normal onset of endogenous *sftpc* , reveals an early, precocious onset of ectopic *sftpc* at NF29 in response to Xenopus Nkx2-1. **(E)** Nkx2-1 is sufficient to induce pulmonary fate in progressively older HG endoderm. Xenopus Nkx2-1 was injected into the HG and activity was induced via DEX treatment at NF 17, NF24, or NF29; embryos were assayed at NF39. Ectopic activation of *sftpc* in the HG is observed in response to all temporal inductions. Abbreviations: lb is lung bud, st is stomach, mg is midgut, and hg is hindgut.

## Description

The amphibian *Xenopus laevis* is a widely used vertebrate model organism with numerous advantages including external embryonic development, ease of experimental manipulations such as targeted microinjection, microdissections, and introduction of small biomolecules readily dissolved in culture media (Edwards and Zorn, 2021). The early *Xenopus* embryo has been extensively fate mapped, which permits targeted microinjection of reagents to specific progenitor populations of organs from all 3 germ layers in the embryo (Moody, 1987; Dale and Slack, 1987). While adult mammalian and amphibian lungs are morphologically very distinct, pulmonary fate induction in the developing foregut as well as early morphogenetic processes of trachea-esophageal septation and lung budding are evolutionarily conserved amongst vertebrates (Zorn and Wells, 2009; Rankin et al *.* , 2015; Rankin et al *.* , 2016; Edwards and Zorn, 2021; Rankin and Zorn 2022b).

The homeodomain TF Nkx2-1 is a master regulator of pulmonary cell fate (Kimura et al *.* , 1996; Minoo et al *.* , 1999), maintaining adult lung function (Boggaram, 2009) and transcriptionally activating lung specific genes *Sftpc* and *Sftpb* (Kelly et al *.* , 1996; Bohinski et al *.* , 1994). In addition, the expression of *Nkx2-1* is used to identify lung specific tissue formed in pluripotent stem cell protocols (Longmire et al *.* , 2012; Rankin et al *.* , 2018) and classify lung cell tumor types (Camolotto et al *.* , 2018).

We generated temporally inducible expression constructs for both *X.laevis* *nkx2-1* and Human *NKX2-1* , fusing the ligand binding domain of the Human nuclear glucocorticoid receptor (GR) to the N-terminus of each; such GR fusions to TFs are widely used in Xenopus to control temporal activity of TFs, as nuclear translocation can be induced by exposure to the synthetic GR ligand Dexamethasone (Dex) which can be added to the embryo culture buffer at any stage of interest (Kolm and Sive, 1995; Sinner et al *.* , 2004; Oropeza and Horb, 2012). In these experiments, messenger RNA (mRNA) encoding the GR-TF fusion is transcribed *in vitro* and then microinjected into the early cleavage stage embryo where it is translated *in vivo;* the GR-TF fusion protein is sequestered in the cytoplasm until embryos are transferred into culture media containing Dex, at which point the fusion protein translocates to the nucleus and exerts activity. We note that while the full length Xenopus Nkx2-1 protein (347 amino acids in length; NCBI Reference Sequence: NP_001079093.1) is shorter than full length Human NKX2-1 protein (401 amino acids; NCBI Reference Sequence: NP_001073136.1), overall the 2 proteins are 70% identical at the amino acid level, with the DNA binding homedomains (human amino acids 194-247) being 100% identical ( **extended data fig.1** ).

In order to determine if NKX2-1 has the ability to reprogram foregut (FG) and hindgut (HG) endoderm into a pulmonary fate, we employed a spatial-temporal gain of function (GOF) assay in Xenopus ( **Fig.1A,B** ). mRNA encoding Xenopus or Human GR-NKX2-1, along with *GFP* mRNA as a lineage trace, was microinjected at the 8-cell stage into dorsal-vegetal (targeting FG) or ventral-vegetal (targeting HG) blastomeres; examination of GFP fluorescence during subsequent development confirmed proper targeting ( **Fig.1B** ). Embryos were transferred into media containing Dex at a number of progressive developmental stages ( **Fig.1A** ), cultured to NF39, and assayed by *in situ* hybridization ( **Fig.1C,D** ). Dex induction of GR-NKX2-1 prior to gastrulation NF10.5 resulted in arrested development / failure of gastrulation. Dex induction of both Xenopus and Human NKX2-1 after gastrulation at NF 13 resulted in the ectopic expression of the pulmonary surfactant genes *sftpc* ( **Fig.1C** ) and *sftpb* (identical to *sftpc* ) in either FG or HG endoderm ( **Fig.1C** ). Interestingly, the foregut/esophagus/stomach TF *sox2* was also ectopically induced, whereas the midgut endoderm marker *ifabp* and mid-hindgut endoderm TF *cdx2* ( **Fig. 1C** ) were both suppressed by Xenopus Nkx2-1and Human NKX2-1. This suggests that NKX2-1 can respecify early post-gastrula foregut and hindgut endoderm into a pulmonary fate. As *nkx2-1* is also endogenously expressed in pharyngeal thyroid endoderm and regulates thyroid development (Small et al *.* , 2000; Hollemann and Pieler 2000; Kurmann et al *.* , 2015; Dame et al *.* , 2017), we examined markers of pharyngeal endoderm, *foxe1* , and thyroid endoderm, *pax2* , however these were unchanged by Nkx2-1 overexpression. We also investigated the temporal onset of ectopic *sftpc* expression when Nkx2-1 was Dex activated at NF13; *sftpc* was detected at NF29 but not earlier ( **Fig.1D** ); this NF29 expression is earlier than the endogenous onset of *sftpc* which normally occurs at NF37/38 in the lung bud (Hyatt et al *.* , 2007; Rankin and Zorn, 2022a), and demonstrates a precocious induction in addition to the ectopic location. It is intriguing why there is a delay between induction of Nkx2-1 at NF13 and activation of *sftpc* (NF29), and this warrants further investigation, perhaps suggesting that alternative transcriptional programs must first be shut off prior to a *sftpc* pulmonary fate being induced.

Endoderm regional identities are progressively determined during Xenopus development (Horb and Slack, 2001; Shifley et al *.* , 2012), and while post-gastrula endoderm is known to still be plastic in potential (McLin et al *.* , 2007; Zhang et al *.* , 2013; Rankin et al *.* , 2018), hindgut fate is specified during gastrulation (Rankin et al *.* , 2018). We thus tested if Nkx2-1 was sufficient to induce pulmonary fate in progressively older endoderm ( **Fig.1E** ). Intriguingly, inducing Xenopus Nkx2-1 activity via Dex treatment at NF 17, NF24, and NF29 all still resulted in ectopic activation of *sftpc* in the HG ( **Fig.1E** ). Similar results were obtained inducing Nkx2-1 in the FG at these older times, suggesting Nkx2-1 has the ability to respecify HG and FG endoderm that is undergoing differentiation/commitment. Future experiments will continue to test progressively older Nkx2-1 induction times.

The ability of individual or combinations of TFs to reprogram one adult or fully differentiated somatic cell type directly into another has garnered great interest for tissue engineering applications aimed to create cell types to treat diseases or investigate mechanisms of regeneration and injury repair. Xenopus has proven a useful model organism to screen the ability of TFs to respecify / reprogram cell fates (Horb et al *.* , 2003; Afelik et al *.* , 2006; Afouda et al *.* , 2018; Oropeza and Horb, 2012; Grand et al *.* , 2023). Our results suggest a single pulmonary TF can respecify developing endoderm: Nkx2-1 can induce pulmonary specific *sftp* expression and suppress alternative fates (midgut, hindgut). Future studies may test if Nkx2-1 is sufficient to induce pulmonary fate in non-endoderm cells (mesoderm and ectoderm). Preliminary targeted injections and analyses suggest Nkx2-1 cannot induce *sftpc* in ectoderm cells, and thus multiple pulmonary or endoderm-specific TFs may be required to convert non-endoderm lineages into pulmonary fate. In addition, our work provides a comparative platform for future studies investigating how mutations in Human *NKX2-1* may affect its transcriptional activity.

## Methods

*Xenopus laevis* care

All experiments involving *Xenopus* were carried out using approved Institutional Animal Care and Use Committee (IACUC) protocols from Bethel University (160302A and R220301) or Cincinnati Children’s Hospital Medical Center (IACUC2022-0026). *Xenopus laevis* were purchased from Xenopus I (Dexter, MI) or Nasco (Fort Atkinson, WI). Induction of egg laying was performed via hormonal injections (Pregnant Mare Gonadotropin (PMG), 50 units 2 days prior and human Chorionic Gonadotropin (hCG), 800 units one day prior to egg laying). Eggs were fertilized with an *in vitro* sperm suspension and kept in a salt solution of one-third (R/3) Marc’s Modified Ringers (MMR) containing 100 ug/ml gentamycin. 8-cell stage embryos with regular cleavage patterns and clear pigment differences were placed in a 4-6% Ficoll solution in R/3 for microinjection. Embryos were staged according to Nieuwkoop and Faber (1994).

Generation of inducible expression constructs

*Xenopus* *laevis* and Human GR-NKX2-1 fusion constructs were produced by PCR amplifying the respective *nkx2-1* genes with primers containing *EcoRV* and *SpeI* recognition sequences. Amplicons were cloned downstream and in frame with the Human GR sequence and 5’ capped mRNA for microinjection was made from linearized plasmid using mMessage mMachine T7 transcription kit (ThermoFisher AM1344).

*In situ* hybridization and photo documentation

Whole mount *in situ* hybridization (WM-ISH) was performed on MEMFA fixed embryos as described (Sive et al *.* , 2000). Anti-sense DIG labeled probes for WM-ISH were created from linearized plasmids containing the cDNA for each of the following genes: *sftpc* , *sftpb* (Hyatt et al *.* , 2007), *sox2* (Ishii Y et al *.* , 1998; Rankin et al *.* , 2015), *ifabp* (Chalmers and Slack, 1998; Shi and Hayes, 1994), *cdx2* (Chalmers et al *.* , 2000; Rankin et al *.* , 2018), *foxe1* (El-Hodiri et al *.* , 2005), and *pax2* (Wang et al *.* , 2011), using T7, T3, or SP6 polymerases and the Roche DIG RNA labeling mix (Millipore Sigma 11277073910) according to the manufacturer’s instructions. Following WM-ISH, embryos were bleached under fluorescent light in a 1% H _2_ O _2_ , 5% Formamide and 0.5X SSC solution followed by refixing in MEMFA. Embryos were transferred to 1X PBS in a 1% 1X PBS agarose dish for scoring and photo documentation on a Ziess Stemi 2000-C stereoscope with a mounted camera.

## Data Availability

Description: Protein alignment of Human, X.laevis NKX2-1. Resource Type: Text. DOI: https://doi.org/10.22002/m6zye-6vx19
